# Demodicidosis Accompanying Acute Cutaneous Graft-Versus-Host Disease after Allogeneic Stem Cell Transplantation

**DOI:** 10.4274/tjh.2018.0057

**Published:** 2018-11-13

**Authors:** Pelin Aytan, Mahmut Yeral, Çiğdem Gereklioğlu, Nazım Emrah Koçer, Nurhilal Büyükkurt, İlknur Kozanoğlu, Hakan Özdoğu, Can Boğa

**Affiliations:** 1 Başkent University Faculty of Medicine, Adana Dr. Turgut Noyan Application and Research Center, Clinic of Hematology, Adana, Turkey; 2 Başkent University Faculty of Medicine, Department of Family Medicine, Adana, Turkey; 3 Başkent University Faculty of Medicine, Adana Dr. Turgut Noyan Application and Research Center, Clinic of Pathology, Adana, Turkey

**Keywords:** Demodex folliculitis, Acute graft-versus-host disease, Post-transplantation

## To the Editor,

**Figure 1:**
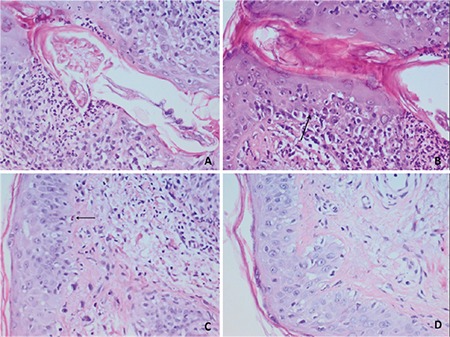
A) Demodex mite; B) Civatte body in the follicular epithelium containing Demodexand lymphocyte exocytosis; C) Civatte body in the epithelium far from the follicle; D) diffuse basal vacuolization in epidermis (periodic acid-schiff staining, magnification 40^x^) (159x119 mm; 72x72 DPI).

A 39-year-old female with acute myeloid leukemia was admitted to our transplantation clinic with face eruption without any pruritus. The eruption had occurred 28 days after she underwent an allogeneic hematopoietic stem cell transplantation (SCT). She was allografted with 6.12x10^6^ non-manipulated CD34+ cells from a fully matched sibling donor after a conditioning regimen including busulfan (12.8 mg/m^2^), fludarabine (150 mg/m^2^), anti-thymocyte globulin (30 mg/kg), and total body irradiation (400 Gy/day). Graft-versus-host disease (GVHD) prophylaxis comprised methotrexate at 12 mg/day for 3 days and cyclosporine A at 75 mg twice daily. No recent changes had been made to the medication. Neutrophil and thrombocyte engraftment both occurred on day 11. The toxicity related to the regimen was mild, being assigned the first grade for oral mucosa according to the Bearman scale [1]. The findings of the physical examination were patchy and confluent erythema of the face, suspicious for cutaneous acute GVHD. There were no other skin changes except that of the palms and soles. Neither intestinal nor hepatic acute GVHD occurred. Laboratory evaluation revealed a white blood cell count of 12,000/µL, a hemoglobin level of 11.5 g/dL, a platelet count of 158,000/µL, and an absolute neutrophil count of 8400/µL. A 4-mm skin punch biopsy was performed [2]. There were lymphocytes and polymorphic neutrophils that attacked hair follicles and two Civatte bodies. Histochemically *Demodex folliculorum *was diagnosed with PAS staining within the hair follicles Figures 1A( and 1B). Even with lymphocytes attacking hair follicles and Civatte bodies suggesting GVHD, *Demodex* folliculitis can mimic acute GVHD (Figures 1C and 1D). Demodicidosis was treated successfully with local 1% metronidazole and 5% permethrin. Methylprednisolone was also administered from the beginning of the symptoms and the dosing was reduced by 8 mg every week. The skin eruptions on the face and the neck resolved on day +52.

*Demodex* folliculitis after allogeneic SCT is seen rarely and, as far as we know, our case is the sixth reported case [3,4,5,6]. The most important differential diagnosis of *Demodex* folliculitis within the first 100 days after allogeneic SCT is acute GVHD. The infestation by *Demodex* sp. can be associated with immune suppression. The differential diagnosis of facial erythema after bone marrow transplantation includes acute GVHD, drug eruptions, systemic lupus erythematosus, viral exanthema, toxic erythema of chemotherapy, drug-induced photosensitivity, and photodermatitis [3]. In our case there were eruptions on the cheek, forehead, and jaw regions, which can be distinguished in both acute GVHD and *Demodex* folliculitis. However, the development of palmar erythema of the upper extremities is not a feature of demodicidosis. As confirmed by pathological examination, there were findings of both acute GVHD (presence of Civatte bodies, lymphocyte exocytosis, diffuse basal vacuolization in the epidermis) and demodicidosis (presence of *Demodex* folliculorum).

It should not be forgotten that GVHD may be associated with demodicidosis and *Demodex* infestation should be remembered in the differential diagnosis of eruptions in patients with hematological malignancies receiving chemotherapy and after SCT. For this reason, when the diagnosis of acute GVHD is ambiguous, an early skin biopsy has to be done after allogeneic SCT because early therapy for a possible *Demodex* infestation would prevent the progression of GVHD.
